# Laparoscopic Repair of Paediatric Indirect Inguinal Hernia: Modified Flip Flap Technique

**Published:** 2020-05-31

**Authors:** A. Garzi, M. Prestipino, E. Calabrò, R.M. Di Crescenzo, M.S. Rubino

**Affiliations:** 1 Division of Pediatric M.I.S. and Robotic Surgery University of Salerno, Italy; 2 Division of Pediatric Surgery A.O. S. Maria della Misericordia Perugia, Italy; 3 Department of Advanced Biomedical Sciences, Pathology Unit, University of Naples Federico II

**Keywords:** Herniotomy, herniorrhaphy, minimal invasive surgery, flip flap technique

## Abstract

In paediatric age, indirect inguinal hernia represents more than 95% of the hernial disease. It is a congenital type, in contrast with adulthood in which acquired forms are more frequently found (1). The laparoscopic correction of indirect inguinal hernia is one of the most common surgeries performed in paediatric age. In recent years, various techniques have been introduced for the videolaparoscopic correction of this disease.

The aim of this study is to provide an assessment of the efficacy and safety of the execution of a modified Flip-Flap technique, using tissue glue for filling of Peritoneal-vaginal duct (DPV), performed in order to ensure greater suture tightness and reduce the incidence of postoperative hydrocele. author1, author2, author 2, author1, author1, author 2

The Authors present a retrospective review of their record of cases, considering a total of 187 patients aged between 18 months and 14 years. For the correction of the hernial defect, the modified VLS Flip-Flap technique was carried out.

The evaluation of safety, efficacy, operating time, relapse rate and development of short-term complications (such as postoperative hydrocele, scrotal hematoma or ecchymosis, atrophy or iatrogenic testicle ascension) was considered in a mean follow-up of 6 months. The Authors suggest that this variant of the peritoneal Flip-Flap technique is simple to perform; its safety, reproducibility and effectiveness is proven and has a percentage of relapses and complications overlapping with the “open” approach and superior to other laparoscopic techniques.

## I. INTRODUCTION

As a part of the nosographic group of disorders of the inguinal canal, indirect inguinal hernia is characterized by a lack or incomplete obliteration of the peritoneal-vaginal duct (DPV). Generally, 60% of hernias occur on the right, 25% on the left and 15% bilaterally (synchronous or metachronous). The M:F ratio is 9–10:1 while there is no evidence of ethnic differences.

In most cases the disease develops within the first year of life, with a major incidence during the first months, but it may be present from birth or develop after the age of 14, especially if there are factors that lead to an increase in the abdominal pressure. The incidence of indirect inguinal hernia in paediatric age ranges from 1% to 5% in births at term, while in the premature child the incidence is markedly higher (reaches 30%) and it is directly proportional to the grades of prematurity. Also, in these patients the percentage of complications is higher, which justifies an early application of surgery (1; 2).

Although the gold standard for correction of the indirect inguinal hernia is still represented by open herniorrhaphy, minimally invasive surgery has proved to be versatile and safe in children. In fact, for the correction of hernias, laparoscopy represents a valid option to the “open” technique (3). Many Videolaparoscopy (VLS) techniques have been introduced over the years; among these, the peritoneal Flip-Flap technique allows a “tension-free” correction of the hernial breach in VLS.

At the beginning, laparoscopic correction of indirect inguinal hernia in paediatric age (El-Gohary, 1997) was performed exclusively in female patients because of the inability to preserve the integrity of the spermatic cord structures in male patients (4). Montupet and Esposito (1998) were the first to perform the laparoscopic approach for correcting inguinal hernias in male children, using a purse-string suture to close the inguinal inner ring (5).

During the correction of the hernial defect, the laparoscopic approach also allows to evaluate, with a sensitivity of 99.4% and specificity of 99.5% (6; 7; 8), the contralateral inguinal canal in order to highlight peritoneal-vaginal duct patency or a hernia still not clinically evident, which is a conditions that several studies over the years have shown to be extremely frequent in the paediatric population, particularly among preterm, with percentages of incidence greater than 50% (9).

## II. MATERIALS AND METHODS

A total of 223 elective laparoscopic repair of indirect inguinal hernia were performed between January 2010 to October 2016 at “A.O.U. Senese di Santa Maria alle Scotte” and the “A.O.U. San Giovanni di Dio e Ruggi d‘Aragona di Salerno”.

All surgeries were performed by a single surgeon using the modified Flip-flap technique. 187 patients (121 males and 66 females) were considered. The patients ranged in age from 18 months to 14 years old. Criteria for enrollment included the diagnosis of an inguinal hernia in patients older less than 15 years of age. Exclusion criteria included patients with incarcerated hernia and strangulated hernia.

We collected data on gender, age, hernia type, single-sided or bilateral hernia, complications, surgical times and recurrence rates. All patients underwent clinical evaluation and ultrasonography preoperatively.

The patients were subjected to a maximum follow-up of 12 months. The follow-up program included postoperative clinical evaluations done by the same surgeon at 7 days, and then 1, 6 months and 12 months after surgery. All intraoperative and postoperative complications were recorded.

All procedures were performed under general anesthesia. The patient is placed supine or in Trendelenburg of 10° or 20°. The first operator is positioned at the patient’s extreme cephalic while the assistant surgeon is on the patient’s side, controlaterally to the hernial defect. A 5-mm trocar with 30° optic is positioned at umbilical level, the pneumoperitoneum is induced up to a pressure of 8–10 mmhg, then 2 2/3-mm operating trocars are introduced at the level of the hips under laparoscopic guide.

When the hernial defect is identified and the presence of a bilateral patency of the inner inguinal ring is excluded, the reduction in the abdomen of the possible contents of the hernial sac is carried out. Then, the peritoneum is cut near the anterior and lateral margins of the hernial defect, thus to create a peritoneal flap large enough to cover it. At this point, in the variant of the technique, before performing the suture of the peritoneal flap, a filling with tissue glue of the DPV is made, in order to ensure a greater tightness of the suture and to reduce the incidence of postoperative hydrocele.

A 4-0 polypropylene suture is inserted directly through the abdominal wall, the peritoneal flap is then reversed medially and fixed with a tight stitch at the intra-abdominal level. The needle is then retracted through one of the work channel and the skin incisions is closed with absorbable suture. This method, therefore, uses a different strategy for surgical therapy of hernias; the hernial defect is not closed by suture as in other methods but covered with a peritoneal flap to obtain a correction “tension-free” which uses intra-abdominal pressure to generate a one-way valve mechanism that closes the hernial sac and keeps it in a collapsed state.

The obvious advantage of this method is the avoidance of manipulation of the structures of the spermatic cord, reducing the risk of damaging and decreasing even post-clinical complication such as the development of hematomas and scrotal edema or long-term complications such as testicular atrophy and infertility (10; 11).

## III. RESULTS

During the study period, 223 elective inguinal hernia repairs were performed on a total of 187 children (151 single-sided and 36 bilateral defects), including 121 (65 %) boys and 66 (35 %) girls. Table 1 summarizes the patients data. There were no intraoperative complications and all 223 procedures were completed successfully. All patients were discharged to home on the same day as the procedure.

The retrospective analysis of the total of 223 procedures performed in VLS with the modified Flip-flap technique, showed that the average surgical times for the correction of the single-sided defects were 32 **±** 7.9 minutes and 46 **±** 8.9 minutes for the correction of bilateral defects. The increase in surgery time, is, however, offset by a reduced incidence of relapses, which in our experience is 0.53% (1 relapse on 187 patients). The mean follow up time was 6 months.

Thanks to the use of tissue glue for the DVP filling, there were no evidence of post-operative hydrocele. However, in the post-operative period, 2% of cases developed a scrotal hematoma and 6% of cases presented a scrotal ecchymosis. Other possible post-operative complications such as iatrogenic testicular ascension, skin injury infection or testicular atrophy were not detected.

## IV. DISCUSSION

For the correction of indirect inguinal hernias, peritoneal Flip-flap technique, is one of the emerging laparoscopic procedures introduced over the years.

In order to support the effectiveness and safety of the modified flip-flap technique in paediatric indirect inguinal hernia, we made a comparison between our observations to previous studies in literature (12; 13; 14; 15; 16; 17; 18; 19; 20; 21; 22; 23; 24).

From the results obtained with the execution of the modified Flip-flap technique, the advantages in common with other laparoscopic procedures, such as the possibility of performing the correction of the hernial defect during the surgery, were demonstrated. In fact, as other laparoscopic techniques, the modified Flip-flap offers the opportunity of a direct intraoperative exploration of the contralateral inguinal canal, thus highlighting the possible cases of contralateral defect and performing the correction during the same operative time (preventing the possible development of metacrone hernias and their complications). In addition, the aesthetic result is excellent and the operating field is virtually free of adhesions in case of relapses and need for re-introduction.

The surgical times related to this technique are therefore greater than the VLS technique of reference (sec. Shier) (19), since there was the need to realize the peritoneal flap before performing the suture. (Table 2)

The correction of the hernial defect is performed without traumatism to the structures of the spermatic cord, since the manipulation of such anatomical structure during the surgery is not necessary, with a drastic reduction of testicular atrophy and iatrogenic ascension of the testicle (25).

In our study the relapses ratio is completely overlapping, and in some cases less than the percentage described in the literature with regard to the open (26, 27) and the VLS technique (Schier), which is around 1,80% (19) (Table 3). In our experience, thanks to the addition of DPV filling with tissue glue, a total absence of postoperative hydrocele was observed (Table 4).

## V. CONCLUSION

Laparoscopic repair of indirect inguinal hernia performed in this experience resulted a safe and effective procedure. The advantages shown by the modified Flip-flap technique include the simple execution of the procedure which allows to obtain a safe closure “tension free” of the DPV, in full respect of the vascular structures and the spermatic cord. Therefore, because of the synergistic action of the peritoneal flap, which acts with a valve mechanism taking advantage of the endo-abdominal pressure, and by adding the filling with tissue glue of the duct, the safety and effectiveness of the procedure is proved. There is also minimal incidence of complications and relapses. However, although the preliminary results are encouraging, it is necessary to carry out a case-control randomized prospective study in order to confirm the validity of the technique with a larger number of recruited subjects and is necessary to perform a longer follow-up to highlight any long-term complication.

## Figures and Tables

**Table 1:** Demographic and clinical data of the pediatric patients with indirect inguinal hernia

Patients data	Modified flip flap technique
Age	18 months – 14 years
Male	121
Female	66
Single sided hernia	151
Bilateral defects	36
Time of surgery single sided	32 ± 7.9 min
Time of surgery bilateral	46 ± 8.9 min

**Table 2:** Average surgical time in monolateral/bilateral correction

SURGICAL TECHNIQUE	AUTHOR	PATIENTS	AVERAGE SURGICAL TIME (MONOLATERAL)	AVERAGE SURGICAL TIME (BILATERAL)

**Modified Flip flap**	Garzi A. (2016)	187	32 ± 7.9min	46 ± 8.9 min

**Extracorporeal**	Tam (2009)	433	23,8min	40,2 min
**Percutaneus IR suturing**	Patkowski (2006)	140	19min	24 min
**Flip flap**	Yip (2004)	32	26±7.9min	38±8.9 min
**Inversion and ligation**	Lipskar (2010)	173	33.9 ± 7.8min	41.9 ± 11.6min
**Resection, no ligation**	Riquelme (2010)	91	40min	51 min
**Intracorporeal**	Giseke 2010	385	26,2min	34,5 min
**Single Port modified**	Li B (2012)	1107	11 (5 – 11) min	20 (14–27) min

**Schier**	Bertozzi M.(2015)	122	29.9±15.9 min	41.5±10.4 min

**Two port laparoscopic**	Dutta S.(2009)	187	15 min	20 min
**DDS**	Becmeur F.(2004)	82	27min	36 min
**Lasso**	Li S.(2014)	207	18.1±5.4min	26.6±4.8min
**Montupet incise and dissect**	Esposito C.(2009)	315	7–30min	(12–49)min
	Shah R. (2013)	155	29min	40 min

**Table 3:** Percentage of relapse: comparison between different technique.

SURGICAL TECHNIQUE	AUTHOR	PATIENTS	RELAPSE (%)

**Modified Flip flap**	**Garzi A. (2016)**	**187**	**0,53%**

**Percutaneus IR suturing**	Patkowski (2006)	140	1,30%
**Extracorporeal**	Tam (2009)	433	0%
**Flip Flap**	Yip (2004)	32	1%
**Inversion and ligation**	Lipskar (2010)	173	0,83%
**Resection, no ligation**	Riquelme (2010)	91	0%
**Intracorporeal**	Giseke (2010)	385	1%
**Single Port modified**	Li B (2012)	1107	0,54%

**Schier**	**Bertozzi M.(2015)**	**122**	**1,80%**

**Two port laparoscopic**	Dutta S.(2009)	187	2,10%
**DDS**	Becmeur F.(2004)	82	0%
**Lasso**	Li S.(2014)	207	0,43%
**Montupet**	Esposito C.(2009)	315	0,61%
**Incise and dissect**	Shah R. (2013)	155	0%

**Table 4:** Post-operative hydrocele incidence

SURGICAL TECHNIQUE	AUTHOR	PATIENTS	POST OPERATIVE HYDROCELE (%)

**Modified Flip flap**	**Garzi A (2016)**	**187**	**0%**

**Percutaneus IR suturing**	Patkowski (2006)	140	3,57%
**Extracorporeal**	Tam (2009)	433	0,47%

**Flip flap**	**Yip (2004)**	**32**	**3,12%**

**Lasso**	Li S.(2014)	186	0%
**Two port laparoscopic**	Dutta S.(2009)	187	1,50%
**DDS**	Becmeur F.(2004)	82	0%
